# Impact of Eukaryotic Translation Initiation Factors on Breast Cancer: Still Much to Investigate

**DOI:** 10.3390/cancers12071984

**Published:** 2020-07-21

**Authors:** Qin Chen, Bo Yang, Norbert Nass, Christoph Schatz, Johannes Haybaeck

**Affiliations:** 1 Department of Pathology, Medical Faculty, Otto-von-Guericke-University Magdeburg, 39104 Magdeburg, Germany; daisychenqin@zju.edu.cn (Q.C.); bo.yang@uk-halle.de (B.Y.); norbert.nass@med.ovgu.de (N.N.); 2 Department of Pathology, The Affiliated Women’s Hospital, School of Medicine, Zhejiang University, Hangzhou 310000, China; 3 Department of Pathology, The Affiliated Hospital of Southwest Medical University, Luzhou 646000, China; 4 Department of Pathology, Neuropathology and Molecular Pathology, Medical University of Innsbruck, 6020 Innsbruck, Austria; christoph.schatz@student.uibk.ac.at; 5 Diagnostic and Research Institute of Pathology, Medical University of Graz, 8010 Graz, Austria; 6 Center for Biomarker Research in Medicine, 8036 Graz, Austria

**Keywords:** eukaryotic initiation factors, translation initiation, breast carcinoma

## Abstract

Breast carcinoma (BC) remains one of the most serious health problems. It is a heterogeneous entity, and mainly classified according to receptor status for estrogen (ER), progesterone (PR) and egf (HER2/Neu), as well as the proliferation marker ki67. Gene expression in eukaryotes is regulated at the level of both gene transcription and translation, where eukaryotic initiation factors (eIFs) are key regulators of protein biosynthesis. Aberrant translation results in an altered cellular proteome, and this clearly effects cell growth supporting tumorigenesis. The relationship between various eIFs and BC entities, as well as the related regulatory mechanisms, has meanwhile become a focus of scientific interest. Here, we give an overview on the current research state of eIF function, focusing on BC.

## 1. Introduction

Worldwide, breast carcinoma (BC) remains one of the most serious health problems. The global annual incidence of reported BC cases is about 1,700,000. It is expected that incidence and mortality rates will increase significantly in the next 5–10 years [1]. Up to now, the median survival time in the advanced stage cohorts is still low (median survival time: 24 months) [2]. In developing countries, the BC rates are disproportionately high, and are estimated to increase to 55% in incidence, and to 58% in mortality, in the next 20 years [3]. BC is a heterogeneous entity, varying in hormone receptor status and expressions of human epidermal growth factor receptor (HER2) [4]. These markers, together with proliferation (as determined by ki67 expression or mitotic counts) and HER2 are used to assign BC to subtypes, and to predict the response to targeted therapies.

Additionally, BC can be classified according to gene expression analysis into four major molecular subtypes, namely luminal A, luminal B, HER2-enriched, and basal like [5,6]. From formalin-fixed paraffin-embedded (FFPE) BC samples, one can directly determine the four original subtypes, by applying a multigene assay i.e., the pam50 gene expression signature [7]. However, in clinical pathological diagnosis, we usually apply immunohistochemistry (IHC) to distinguish the four subtypes, by determining the status of the estrogen receptor (ER), progesterone receptor (PR), HER2 and Ki67 in BC tissue. The different expression status of these four parameters can used to be catalogued the subtypes. Luminal A-like subtype (approximate 40%) displays ER+ or PR+, or both, HER2− and low Ki67; luminal B-like subtype (approximate 40%) shows ER+ or PR+, or both, HER2− and high Ki67; HER2 subtype is divided into non-luminal (HER2+ and ER− and PR− (or luminal (HER2+ and ER+ or PR+, or both); basal-like subtype (HER2− and ER− and PR−, also called triple negative breast cancer). Up to now, at least ten different molecular subtypes have been identified using gene copy number and expression analyses [8]. These molecular subtypes are then used to guide systemic therapy for BC.

BC subtypes differ markedly in prognosis and in the therapeutic treatment. BC patients are usually treated by combination therapies of surgery, endocrine therapy, chemotherapy and radiotherapy. ER-positive BC patients are usually treated with five years of adjuvant endocrine therapy (Tamoxifen for premenopausal women or aromatase inhibitor or a combination of both after menopause). For preventing relapse in hormone-positive BC, patients might accept an extended endocrine therapy beyond five years [9]. Targeted therapy has substantially contributed to progress in BC therapy. Correspondingly, improved personalized treatments have minimized the side effects and improved overall survival rates. However, there is an annual increase in resistance rates to targeted drugs, depending on intra-tumor heterogeneity, adaptive processes and the patient’s genetic variation [3]. Even when targeted therapeutic strategies are applied, all breast cancer types might confront the oncologist with developing drug resistance. BC could resist multiple treatment strategies, such as chemotherapy, endocrine therapy or monoclonal antibody therapy. Hence, the current available targeted therapies are often limited.

Eukaryotic gene expression is mainly regulated at the level of transcription and translation. The deterioration of protein biosynthesis, divided into initiation, elongation, termination and ribosomal recycling, leads to an abnormal cellular proteome that can cause uncontrolled cell growth, as far as carcinogenesis is concerned [10]. Of the four stages of the translational cascade, initiation is the pivotal rate-limiting step. The eukaryotic initiation factors (eIFs) act as protein complexes called eIF1, eIF2, eIF3, eIF4, eIF5 and eIF6.

Indeed, previous research has shown that the dysregulation of several eIFs are related to carcinogenesis and cancer progression [11,12,13,14,15]. In this review, we will emphasize the contribution of eIFs to breast carcinogenesis, and provide a theoretical basis for new-targeted therapies.

## 2. Eukaryotic Translation Initiation Factors in Breast Cancer

### 2.1. Eukaryotic Translation Initiation Factor 1 (eIF1)

*eIF1* was identified as product of a damage-induced cDNA termed A121 [16], indicating that genotoxic stress could modulate translation initiation via *eIF1*. eIF1 is composed of 113 amino acid (AA) and highly conserved from bacteria to humans. eIF1a is a 144 AA long protein encoded on chromosome X. By changing the structure of the mRNA-binding of initiation complex to the first AUG initiation codon, both of them are required in translation initiation and mRNA screening. The function of eIF1 and eIF1a is synergistic; both are required for 48S complex assembly and binding at the initiation codon [17]. Little is known about eIF1 and eIF1a in the carcinogenesis of BC.

EIF1AX mutations are common in advanced thyroid cancers, accompanied by a simultaneous occurrence of RAS mutations. The cooperation of EIF1AX and RAS mutations can induce the formation of tumors in mice [18]. The co-occurrence and subsequent co-expression of mutant NRAS and EIF1AX promoted proliferation and survival in ovarian low grade serous carcinomas [19]. In breast cancer, about 2.4% and 0.9% of cases were reported to have genomic alterations for eif1 or eif1ax, respectively [20].

### 2.2. Eukaryotic Translation Initiation Factor 2 (eIF2)

**Figure 1:**
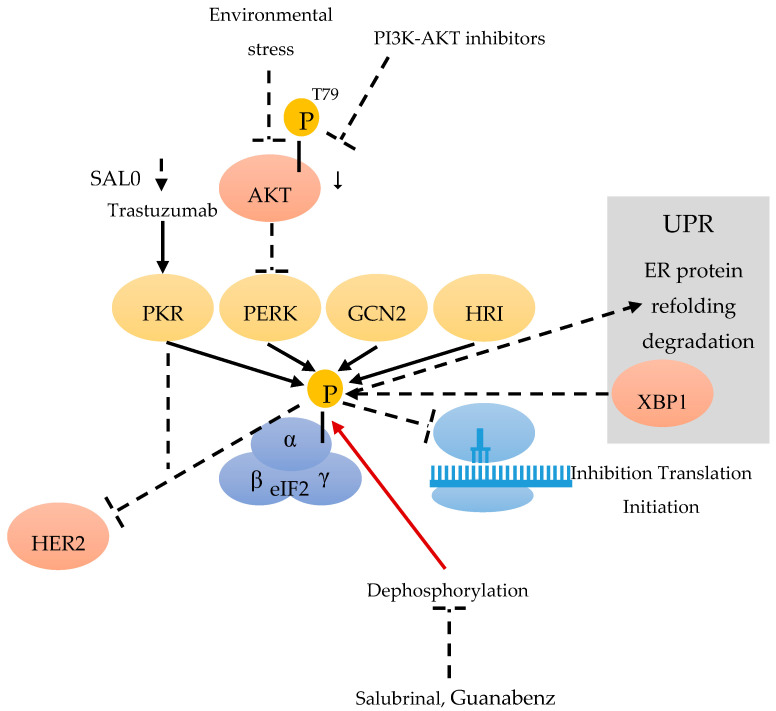
Eukaryotic initiation factor 2 (eIF2) consists of the α, β and γ subunit. The kinases double stranded RNA-activated kinase (PKR) [22], PKR-like endoplasmic reticulum kinase (PERK), general control non-repressed 2 (GCN2) [23] and heme-regulated inhibitor (HRI) [24] can phosphorylate eIF2α during a stress response. Environmental stress and PI3K-AKT inhibitors are able to down-regulate AKT-p-T799 and to activate PERK [13]. SAL003 can improve the efficiency of Trastuzumab, and Trastuzumab can stimulate PKR [25]. eIF2α-p leads to an inhibition of the translation initiation [26] and can inhibit HER2+. Stress induces the expression of X-box binding protein 1 (XBP1) of the unfolded protein response (UPR) [29], by phosphorylating eIF2α, which results in protein refolding and degradation. Salubrinal and Guanabenz can inhibit the dephosphorylation of eIF2α-p.

eIF2 is composed of three subunits: eIF2α, β and γ (Figure 1). DNA damage, oxidative stress, endoplasmic reticulum (ER) stress, and stress occurring in the tumor microenvironment, can lead to the phosphorylation of the eIF2α subunit on Ser51, thereby reducing the level of active eIF2 [21]. Although multiple stresses can result in eIF2α phosphorylation, the cellular outcome is not always the same. Phosphorylation of eIF2α (eIF2α-p) is catalyzed by four kinases (eIF2AKs): double stranded RNA-activated kinase (PKR) [22], PKR-like endoplasmic reticulum kinase (PERK), general control non-repressed 2 (GCN2) kinase [23] and heme-regulated inhibitor (HRI) [24]. All of the above are referred to as the integrated stress response (ISR) (Figure 1). Increased phospho-eIF2α not only leads to the mRNA translation initiation inhibition, but also facilitates the translation of selected mRNAs, which encode proteins participated in the process of adaptation to stress [25].

The PKR/eIF2α-P displays anti-tumor effects and can suppress HER2+ BC growth in mice [26] (Figure 1). Trastuzumab can induce the PKR/eIF2α-P and its downstream anti-tumor pathways, in sensitive but not resistant HER2+ BC. For the overall survival rate of HER2+ BC patients treated with Trastuzumab-based chemotherapy, an increased level of eIF2α-P can act as an independent positive prognostic marker [25]. As the stimulation of eIF2α phosphorylation by the phosphatase inhibitor (SAL003) can improve the efficacy of Trastuzumab, it might be a potential biomarker for its effectiveness [25] (Figure 1).

Previous research has indicated that diet-driven IFN-γ could promote the malignant transformation of primary bovine mammary epithelial cells through accelerating arginine depletion [27]. Subsequently, Ren et al. reported that, on account of arginine addition, eIF2α was involved in the inhibition of malignant transformation of mammary epithelial cells. The NF-κB-GCN2/eIF2α pathway can alleviate malignant transformation in IFN-γ-induced mammary epithelial cells through reducing the ability of cell proliferation, migration and colony formation [26].

As a highly conserved pathway, the unfolded protein response (UPR) allows cells to react to ER stress imposed by environmental forces [28]. By transcriptional activating XBP1, the UPR allows cells to adapt to environmental stress through the restauration of protein folding homeostasis, by phosphorylating eIF2alpha and increasing the capacities of ER protein-folding and degradation. The UPR includes three parallel pathways: PKR-like ER kinase (PERK)–eIF2α pathway; inositol-requiring protein 1α (IRE1α)–X-box binding protein 1(XBP1) pathway (Figure 1); and ATF 6α pathway [29]. The PERK–eIF2α pathway in tumors may participate in tumor cells, in deciding whether survival or apoptosis is caused by ER stress, also promoting or inhibiting malignant transformation [30]. In recent research, G-protein-coupled estrogen receptor (GPER)-specific agonist G1 could induce ER stress in the estrogen receptor positive BC cell line (MCF-7). The mechanism was correlated with G1-induced Ca^2+^ efflux from the ER, which results in the activation of UPR. The pro-survival UPR signaling was activated by GRP78 and a translational decrease was indicated by eIF2α phosphorylation. The pro-death UPR signaling was also activated, indicated by PERK-p induced JNK-p and IRE1α-p, and finally triggering the phosphorylation of CAMKII. Ultimately, the ER Ca^2+^ decrease is responsible for G1 induced cell death by pro-death UPR signaling [31].

Stress can promote the expression of MBP-1 through the AKT/PERK/eIF2α signaling pathway. During this signal transduction, PERK is negatively regulated by AKT-dependent T799-phosphorylation. Inactivated PERK does not phosphorylate eIF2α [13] (Figure 1), which consequently leads to a decrease of phospho eIF2α. Treating cells with PI3K-AKT inhibitors can induce ER stress, resulting in AKT-p down-regulation and the activation of PERK kinase and the phosphorylation of eIF2a [13]. All the points mentioned above support the connection between the active/inactive states of AKT, PERK-mediated eIF2a phosphorylation and MBP-1 expression [32,33].

The eIF2α-mediated Rac1 pathway also participates in proliferation and survival of tumor cells. Blocking eIF2α dephosphorylation can alter cell proliferation and differentiation via the regulation of Rac1 [13]. The phosphatase inhibitors salubrinal and guanabenz, which cause increased phospho-eIF2α, can inhibit human triple negative BC cell lines (4T1 and MDA-MB-231), in terms of proliferation, cell survival, invasion and motility. An in vivo assay also revealed that the subcutaneous administration of salubrinal could reduce the tumor volume and weight induced using BALB/c mice injected with 4T1 cells. These results indicate that these phosphatase inhibitors can decrease the tumor growth of BC cells through the eIF2α-mediated Rac1 pathway. Because of an additional inhibition of bone resorption by salubrinal and guanabenz, these results suggest that eIF2α-mediated Rac1 regulation can be useful for suppressing the growth and metastasis of BC [13] (Figure 1).

### 2.3. Eukaryotic Translation Initiation Factor 3 (eIF3)

**Figure 2:**
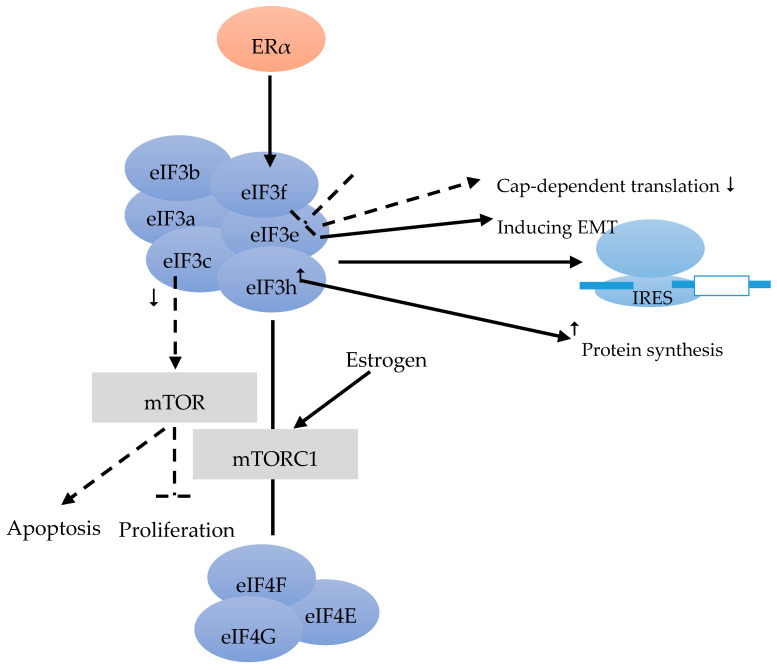
eIF3 consists of the conserved eIF3a, eIF3b, eiF3c and the less conserved eIF3e, eIF3f, eIF3h subunits [17]. eIF3 is required for IRES [34]. eIF3e induces epithelial-to-mesenchymal transition (EMT), and a down-regulation leads to a decrease of cap-dependent translation [36]. Estrogen alpha (ERα) controls eIF3f at transcriptional and translational level [37]. mTORC1 pathway could be activated by estrogen, and enhances the binding of eIF3 to the EIF4F complex [37]. Decreased eIF3c can suppress the proliferation, and can lead to apoptosis via the mTOR pathway [38]. An overexpression of eIF3h leads to an increase in protein synthesis [39].

The eIF3 complex comprises at least six core subunits, of which 50% are well conserved in evolution (eIF3a, eIF3b, eIF3c), whereas 50% are not (eIF3e, eIF3f, eIF3h) [17] (Figure 2). The eIF3 complex takes over different tasks that are not specifically designated to its single subunits during translation initiation. Furthermore, eIF3 is also important and required for IRES (intergenic ribosome entry site)-dependent translation initiation [34] (Figure 2).

Moreover, 37% of BC showed reduced eIF3e expression compared to the stromal tissue [35]. Besides inducing epithelial-to-mesenchymal transition (EMT), the down-regulation of eIF3e expression was found to result in a repression of cap-dependent translation (Figure 2). The combination of transcriptional and translational regulation is involved in EMT in cells with reduced eIF3e expression [36].

According to Cuesta et al. [37] ER-positive BC cells have a lower level of eIF3f, compared with ER-negative cells. The authors stated that the low eIF3f level was required for proliferation and survival of ER-positive cells such as MCF-7. The eIF3f expression is tightly controlled by ERα at the transcriptional and translational level (Figure 2). Estrogen can activate the mTORC1 pathway to regulate translation and to enhance the binding of eIF3 to the eIF4F complex (Figure 2). Finally, it facilitates the assembly of 48 S pre-initiation complex and protein synthesis. Referring to the proliferation and survival of ER-positive BC cells, the estrogen-ERα-mediated control of eIF3f expression deserves to be a scientific focus. This might provide a rational for new therapies for ER-positive BC [37]. Moreover, decreased eIF3c can also suppress proliferation and stimulate apoptosis in BC cell line via the mTOR pathway [38] (Figure 2).

Earlier studies found that the elevation of eIF3h could stimulate protein synthesis. Lili Zhang et al. investigated the effect of over-expression of eIF3h on protein synthesis, to figure out the function of eIF3h in different BC cell lines. The tight correlation of high level of eIF3h with the stimulation of protein synthesis suggested that eIF3h plays an important role in regulating protein translation. Hence, like eIF4E and eIF4G, eIF3h also affects protein synthesis and tumor aggressiveness [39] (Figure 2).

### 2.4. Eukaryotic Translation Initiation Factor 4 (eIF4)

The recruitment of ribosome to mRNA templates is the key rate-limiting step of translation initiation, which is mediated by eIF4F, a heterotrimeric complex [40] (Figure 4).

**Figure 3:**
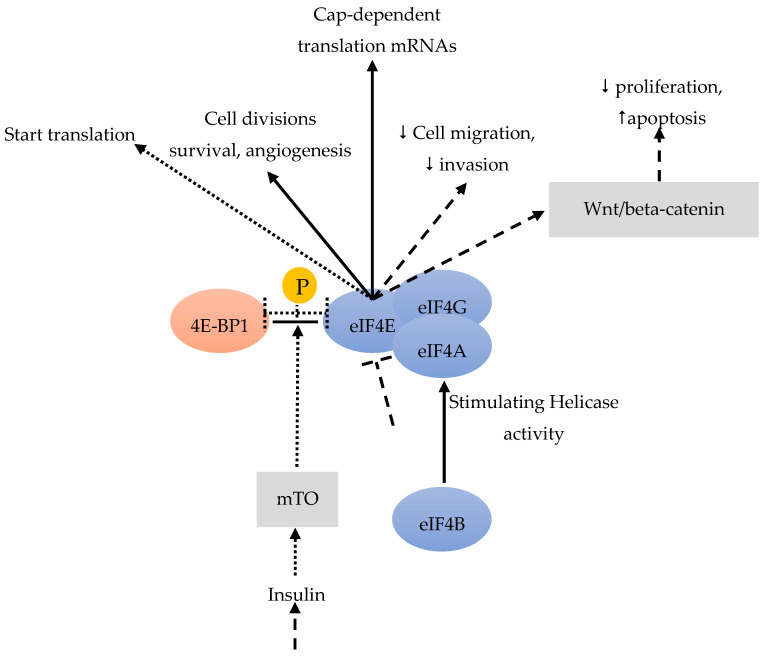
eIF4B can stimulate eIF4A [40]. eIF4E is involved in cap-dependent translation, in cell divisions, survival and angiogenesis [44]. A knockdown of eIF4E leads to an activation of the Wnt/beta-catenin pathway, with anti-proliferative and pro-apoptotic effects [49]. A knockdown of eIF4E also reduces cell migration and invasion [12]. Insulin and other growth factors could stimulate 4E-BP1 [46]. mTOR phosphorylates 4E-BP1 which frees eIF4E [46].

Acting as RNA-dependent ATPase and bidirectional RNA helicase, eIF4a is a member of the DEAD-box family [40]. The eIF4a helicase activity can be stimulated by eIF4b (Figure 3). Residual BC stem cells become the reason of relapse after chemotherapy. Sridharan et al. [41] found that Rocaglamide A (RocA) was effective against triple-negative BC cells by targeting the eIF4A bound to the oncogenic mRNAs, that requires its helicase activity during translation. In the process of RocA effectively against BC stem cells, eIF4A act as an actionable molecular target in both BC stem cells and other tumor cells. Consequently, anti-eIF4A inhibitors could potentially be combined with chemotherapy, radiotherapy and/or immunotherapies.

In BC and other cancers, eIF4E is frequently over-expressed in the very early stage, especially in the pre-invasive stage (such as carcinoma in situ) and associated with the malignant progression of a high tumor grade. eIF4E is also frequently over-expressed in at least 50% of BC [42]. The over-expression and activation of eIF4E are connected with tumor formation, metastasis and increased tumor invasion [43]. eIF4E is involved in the cap-dependent translation of specific mRNAs, including genes important for cell divisions, survival and angiogenesis [44] (Figure 3). Importantly, tumor cells with over-expressed eIF4E may develop an addiction to eIF4E, making these cells vulnerable to eIF4E inhibition, whereas non-malignant cells are resistant. If eIF4E cannot be phosphorylated by MAP-kinases or TGFβ, metastasis is reduced [45]. Knockdown of eIF4E has indeed reduced BC cell migration and invasion [12] (Figure 3). All of these results suggest that eIF4E could drive metastasis.

**Figure 4:**
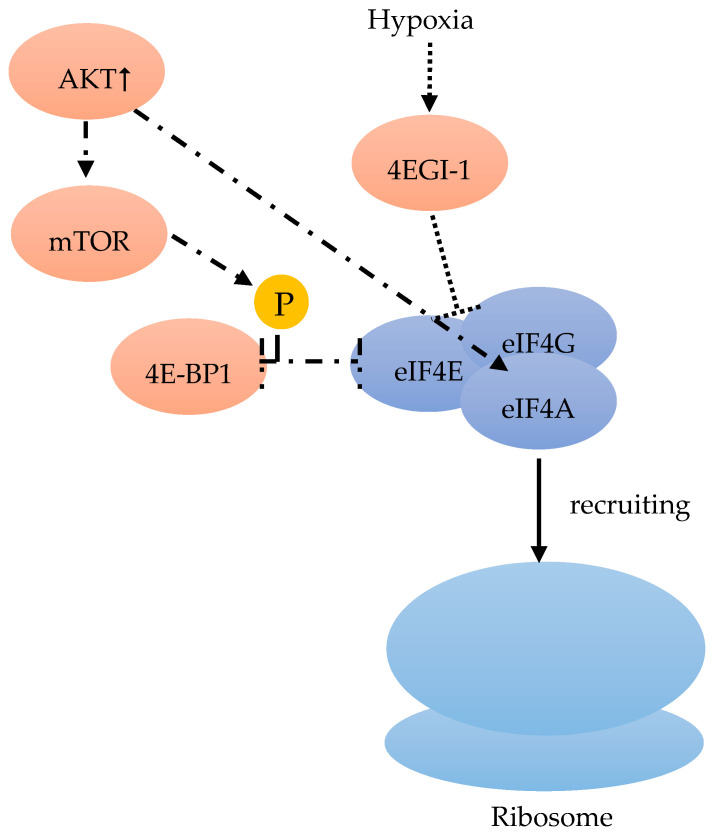
eIF4F consists of eIF4A, eIF4E and eIF4G. eIF4F recruits the ribosome [40]. mTOR phosphorylates 4E-BP1, which leads to a release of eIF4E [46]. Released eIF4E can start translation with interaction in combination with the complex [46]. Hypoxic carcinoma cells are more sensitive to the inhibitor 4EGI-1, which inhibits eIF4E-eiF4G [48]. An increase of AKT stimulates the mTOR dependent phosphorylation of 4E-BP1, and regulates eIF4A availability [56].

Among the three 4E-BPs, the well-studied 4E-BP1 particularly responds to stimulation by insulin and other growth factors, thereby regulating eiF4F (Figure 3). When cells are subjected to such stimuli, mTOR is activated and phosphorylates 4E-BP1, which promotes the release of eIF4E (Figure 4). This free eIF4E can then interact with the eIF4F complex to start translation [46] (Figure 3, Figure 4).

Zindy et al. [47] reported that targeting eIF4E by antisense inhibition can remove the eIF4A helicase from the eIF4F complex, thus delaying BC cell growth in xenograft models. Targeting eIF4F to block BC progression is therefore a rationale choice [12].

**Figure 5:**
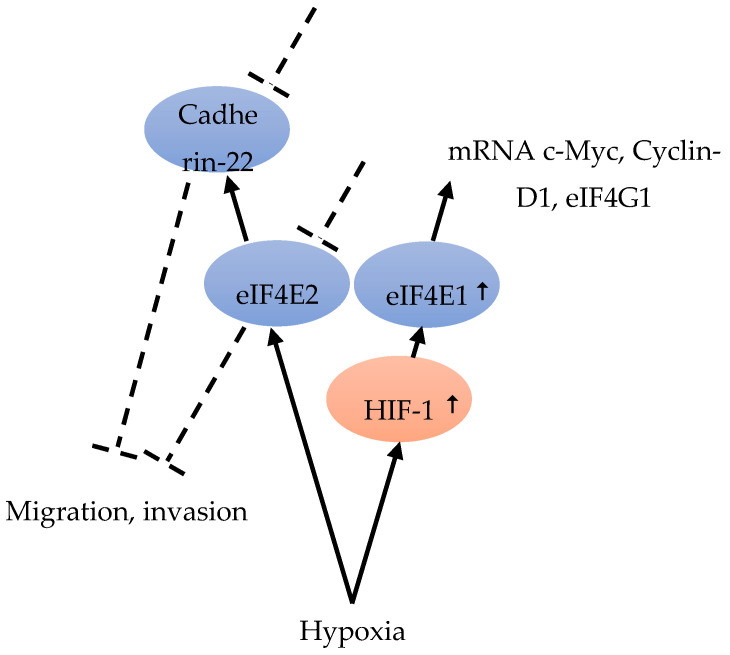
Hypoxia is able to up-regulate eIF4E2 and eIF4E1. eIF4E1 is HIF-1-dependent and HIF-1 increased the expression of eIF4E1 [48]. eIF4E1 leads to an increase in c-Myc, Cyclin-D1 and eIF4G1 mRNA [48]. eIF4E2 under hypoxia regulates Cadherin-22. Blocking eIF4E2 or Cadherin-22 under hypoxia can impair cell migration and invasion [15].

Although hypoxia can up-regulate the expression of eIF4E1 and eIF4E2, only eIF4E1 expression was HIF-1 dependent (Figure 5). In hypoxic tumor cells, HIF-1 up-regulated eIF4E1, resulting in the enhanced translation of certain mRNAs encoding proteins, such as c-Myc, Cyclin-D1, and eIF4G1, which are important for BC cell three-dimensional structure growth (Figure 5). Hypoxic carcinoma cells were more sensitive to the eIF4E-eIF4G interaction inhibitor 4EGI-1, compared to normoxic carcinoma cells, indicating the important role of eIF4F-controlled translation initiation under hypoxia [48] (Figure 4). The intercellular adhesion molecule, cadherin-22, is up-regulated in hypoxia in an mTORC1-independent translational control via eIF4E2 (Figure 5). Under hypoxia, blocking eIF4E2 or cadherin-22 can significantly impair the ability of MDA-MB-231 BC cells to migrate and invade [15] (Figure 5).

**Figure 6:**
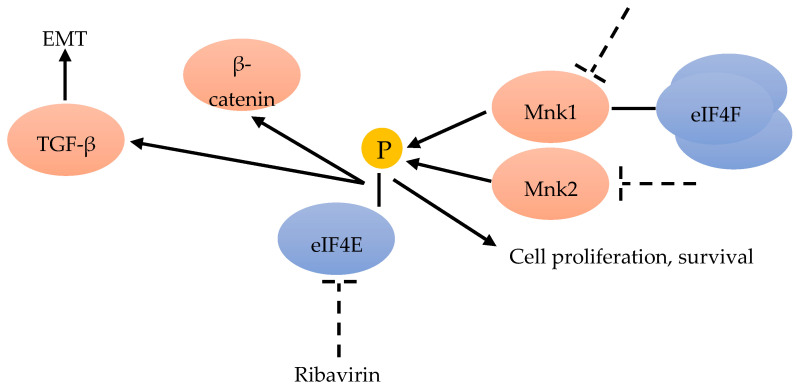
Mnk inhibitors can prevent the phosphorylation of eIF4E and β-catenin [49]. EMT could be the result of TGF-β translational activation through eIF4E phosphorylation [45]. Ribavirin could inhibit eIF4E [61]. Mnk1 is associated with eIF4F and can phosphorylate eIF4E, as well as Mnk2 [67].

Advanced BC is often resistant to chemotherapy. Phospho-eIF4E significantly correlates with a worse clinical outcome. Knockdown of eIF4E enhances the anti-proliferative and pro-apoptotic effects of chemotherapeutic drugs in BC cells by activating the Wnt/beta-catenin signaling pathway (Figure 3). MAPK-interacting kinase (MNK) inhibitors can prevent chemotherapeutic drug-induced eIF4E phosphorylation and β-catenin activation, suggesting that MNK-eIF4E-beta-catenin can restore the sensitivity to chemotherapy (Figure 6). These results highlight the therapeutic value of inhibiting MNK to overcome chemo-resistance in BC chemotherapy [49].

Male BC accounts for less than 1% of all BC cases. Studies on male BC are relatively rare. Much of the published research has focused on comparisons with female BC, and even treatment is also deduced from females [50]. Compared to female BC, male BC is more likely to be estrogen receptor positive (92% vs. 78%), and is different with regards to genetic, transcriptional and protein expression profiles [50]. In female BC, eIF4E and 4E-BP1-p are frequently expressed at higher levels, compared with normal breast tissue, and a higher level of eIF4E is associated with a worse prognosis [51,52]. There are positive associations with grade, lymph node metastasis status and disease recurrence [53]. eIF4E activity in female BC is known to be down-regulated by mTOR high expression and phosphorylation of its binding protein 4E-BP1. The importance of the eIF4E pathway in male BC is still unknown. As phosphorylated 4E-BP1 indicates prognosis in male BC, therapies targeting its upstream kinase (mTOR) can be useful [50].

In women, a combined analysis of the expression of eIF4E, 4E-BP1 and 4E-BP1-p can indicate eIF4E activity and predict BC survival [54].

eIF4E in tumor has therefore become an anticancer drug target. Although inhibiting mTORC1 or mTOR kinase modifies eIF4E activity, this approach is different to targeting eIF4E or eIF4E-BPs directly (Table 1). In male BC, however, little is known about the prognostic connection of eIF4E and the 4E-BPs, so there is no indication that eIF4E-targeted therapies might be considered for this disease [50].

mTOR can regulate the eIF4F complex activity through the phosphorylation of 4E-BP. In BC genetic alteration or epigenetic changes can frequently influence the PI3K/Akt/mTOR pathway, which results in the constitutive pathway activation. In in vitro and in vivo experiments, the activated PI3K/Akt/mTOR pathway is always associated with tumor initiation, and maintenance by unregulated translational control [55]. On the one hand, the increased phosphatidylinositol 3-kinase (PI3K)/Akt results in an extended 4E-BP1 mTOR-dependent phosphorylation, followed by an increase of the dissociation from eIF4E, thus stimulating the formation of eIF4F complex [40]. On the other hand, the PI3K/Akt pathway can also regulate the eIF4A availability in the eIF4F complex assembly [56] (Figure 4). Hence, either through the use of analogs or mTOR-kinase inhibitors (KIs) to inhibit mTOR, or through PI3K/mTOR-kinase inhibitors to suppress PI3K/mTOR activity, an anticancer effect might be achieved [57].

**Figure 7:**
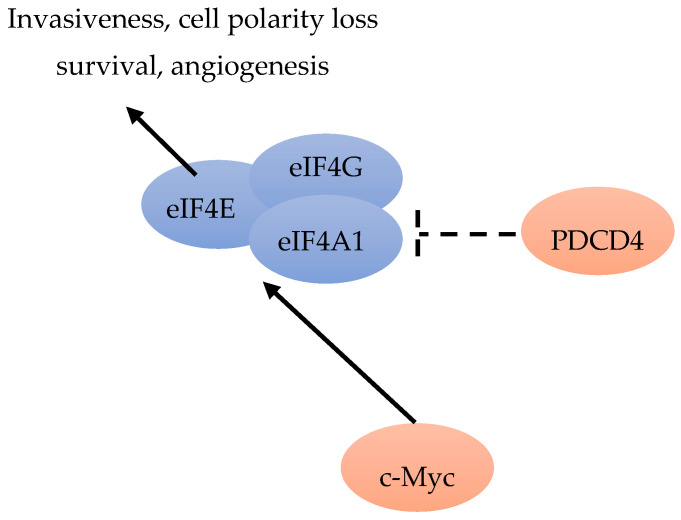
c-Myc can activate eIF4E, eIF4G and eIF4A1 [59]. PDCD4 is an inhibitor of eIF4A1 [64]. Increased levels of eIF4E could lead to invasiveness, cell polarity loss, angiogenesis and cell survival [14].

According to Liu et al., the amplification of *MYC* can lead to PI3K-independent BC cell survival and resistance to PI3K inhibitors [58]. The role of *c-Myc* during cell growth and proliferation, accompanied by increased eIF4F activity, indicated a mechanism involving *c-Myc* and eIF4F. Liu et al. found that c-Myc could directly activate the transcription of three subunits of eIF4F (Figure 7). Increased eIF4F specifically stimulates *c-Myc* mRNA translation. Although *c-Myc* translation occurs through cap-dependent and IRES-mediated translation, a part of c-Myc translation is eIF4F responsive. Hence, all the results suggested that there was an interaction circle between c-Myc and eIF4F, connecting transcription and translation. As the transcriptional target of MYC, eIF4E, eIF4AI and eIF4G may contribute to MYC expression in the drug-resistant background [59].

The epithelial-to-mesenchymal transition (EMT) is important for metastasis and invasion. One important EMT regulator is the cytokine-transforming growth factor beta (TGF-β) [60]. TGF-β can induce EMT by translational activation via another non-canonical TGF-β signaling pathway through eIF4E phosphorylation [45] (Figure 6). The antiviral drug ribavirin has been shown to exhibit antitumor activity linked to the inhibition of eIF4E in patients [61] (Figure 6). Ribavirin, one of antiviral drug, has been demonstrated to inhibit eIF4E by competing with the 7-methylguanosine mRNA cap, and has suppressed proliferation and the clonogenic potential of BC cells in vitro with the elevated levels of eIF4E [42,61,62]. Hence, ribavirin was considered to be a remarkable inhibitor of primary tumor growth in an eIF4E-dependent manner, in vivo and in vitro [63].

The human mRNA DeXD/H-box helicases are molecular motors required in RNA metabolism. Among them, the most common helicase is eIF4A, required during the protein synthesis initiation phase. Immunohistochemical analysis was performed on over 3000 breast tumors. The results indicated that eIF4A1, eIF4B and eIF4E are independent worse prognosis predictors in ER-negative disease. The eIF4A1 inhibitor PDCD4 can improve outcome in ER-positive BC patients (Figure 7). The modulation of eIF4A1, eIF4B and PCDC4 expression in MCF7 cells can inhibit BC cell growth. Immunohistochemical tests are promising tools for testing tumors sensitive to anti-helicase therapies [64].

mRNA translation includes ribosomes recruitment to the capped mRNAs by eIF4F, including initiation factor eIF4E, RNA helicase eIF4A, and scaffolding protein eIF4G [65]. The increased eIF4E level can selectively stimulate translation of “eIF4E-sensitive” mRNAs [65]. By increasing the translation of IRES-containing p120 mRNAs, over-expressed eIF4G can promote formation of inflammatory BC emboli [10]. In precursors of neoplasia, such as atypical hyperplasia or carcinoma in site, increased levels of eIF4E can promote a series of events, including cell invasiveness acquisition, cell polarity loss and cell survival increase (Figure 7), by facilitating the translationally regulated assembly of TGFβ receptor signaling complexes. Increased levels of eIF4E in early neoplastic cells can promote the translation of integrin β1, possibly including metalloproteinase (MMP)-2 and -9 mRNAs. They may transform non-transformed cells sensitive to stimulation by low level of activated TGFβ, via formation of pre-loaded TGFβ receptor signaling complexes, and possibly evolve to a pro-neoplastic phenotype [14].

As a cap-dependent translation rate-limiting factor, eIF4E plays an important role in some weak mRNA translation, such as cyclin D1, Bcl-2, MMPs and VEGF [66]. eIF4E facilitates the efficient mRNA translation initiation by binding to the 5′-cap of eukaryotic mRNAs [67]. A connection between the elevated level of eIF4E and cell transformation was observed in tumors of the breast, bladder, colon, head and neck, lung, thyroid and in lymphoma [68]. The elevated level of eIF4E is also critical for BC progression and angiogenesis [69,70]. All the data presented above suggest that targeting MAPK/Mnk signaling and blocking eIF4E protein translation may constitute a promising strategy for treating BC [71].

eIF4E has also been identified as a poor prognostic marker in several retrospective and prospective studies on BC [72,73]. Several recent studies have identified the oncoprotein eIF4E and its phosphorylated forms over-expressed in BCs [68]. Phosphorylation of eIF4E at Ser209, in response to various extracellular stimuli, increases the affinity of eIF4E for the mRNA 5′ cap, and influences its entry into the translation initiation complex. The best candidate for eIF4E phosphorylation is the mitogen-activated protein kinase (MAPK)-activated protein kinase Mnk1. Mnk1 physically associates with eIF4F, and can directly phosphorylate eIF4E (Figure 6). Mnk2 is also recognized to directly phosphorylate eIF4E, but to a lesser extent (Figure 6). Mnk-mediated eIF4E phosphorylation favors the mRNA translation of proteins involved in tumor cell proliferation and survival [67]. While the Mnk function and eIF4E phosphorylation are considered necessary for malignant transformation, Mnk-mediated eIF4E phosphorylation has proven not to be essential for the normal development of the organism [74]. Thus, targeting the Mnk/eIF4E pathway has become an attractive strategy for BC therapy [71].

### 2.5. Eukaryotic Translation Initiation Factor 5 (eIF5)

During the translation initiation process, two GTPases (eIF5 and eIF2) play key roles. eIF5 is an essential core protein in translation initiation. Upon correct matching to the start codon AUG, GTP is hydrolyzed by eIF2 in an eIF5-dependent manner. eIF5 has an independent GDP dissociation inhibitor activity [75]. eIF5 has been reported to restrain eIF2B, which can inhibit the exchange of guanine and nucleotide and support the following eIF2 recycling. The eIF5 C-terminal end is involved in building the core of the ribosomal pre-initiation complex. It can interact on several sites with other translation initiation factors, such as eIF1, eIF2, eIF3 and eIF4g.

During translation initiation, the G-protein eIF5B takes control of subunit integration with the help of eIF1A. The interactions between eIF1A and eIF5B continuously cycle during translation initiation [76,77].

During the protein translation cycle, eIF5A, as one of many auxiliary proteins, participates in stimulating specific processes. eIF5A can stimulate formation of the first peptide bond between Met-tRNA and puromycin [78]. eIF5A is not considered an initiation factor, as it is homologous to the prokaryotic elongation factor EF-P, and is involved in the elongation and termination of translation, as well [78].

The roles of eIF5, eIF5A and eIF5B in BC biology have not yet been well described. After receiving everolimus and other PI3K/mTOR inhibitors, these biomarkers were immunohistochemically assessed in male BC. After treatment with everolimus, a marked reduction in eIF4E and eIF5 expression was observed in male BC patients with extended survival. Therefore, in male BC patients, the inhibition of eIF4E and eIF5 expression could be of clinical value [79].

### 2.6. Eukaryotic Translation Initiation Factor 6 (eIF6)

**Figure 8:**
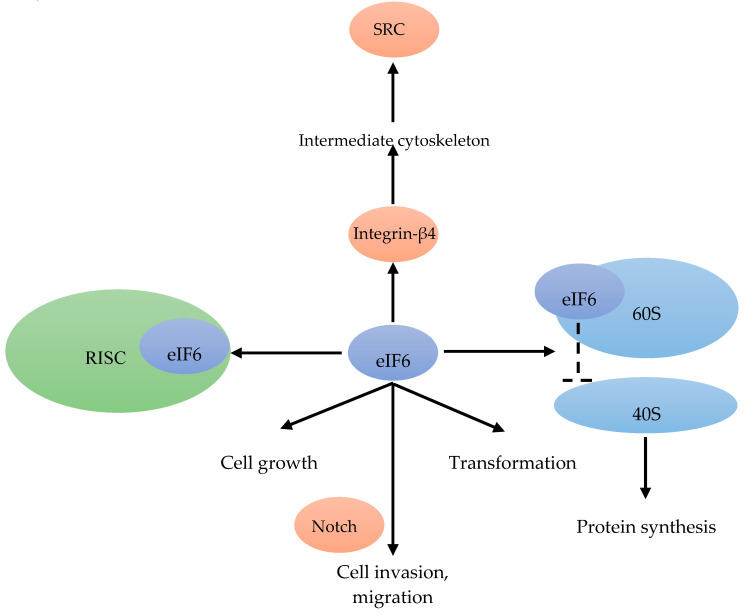
eIF6 binds to the 60S ribosomal subunit, and inhibits the interaction between the 40S and 60S subunit [80]. eIF6 is also associated with the RISC [81]. eIF6, in combination with Notch, mediates cell invasion and migration [80]. eIF6 is able to link integrin-β4 to the intermediate cytoskeleton [84] resulting in the activation of SRC [82]. Cell growth and transformation are associated with eIF6 [81].

In translation initiation, eIF6 acts as a ribosomal anti-association factor. 70% of eIF6 is localized in the cytoplasm and 30% in the nucleus. eIF6 binds to the 60S ribosomal subunit in the nucleus (Figure 8). When bound to eIF6, the interaction between the 60S and 40S subunit is inhibited, and the translation initiation is blocked [80] (Figure 8). The translation regulation by eIF6 was also reported as a rate-limiting step [81]. eIF6 is also part of a multi-protein complex associated with the RNA-induced silencing complex, which can regulate miRNA activity (Figure 8). There, eIF6 interacts with pre-ribosomal particles, performing a critical role in the assembly of the 60S ribosome [80]. Hence, the expression of eIF6 is also involved in cell growth and transformation [81] (Figure 8).

In the highly proliferative and therapy resistant luminal B BC subtype, eight genes (*FGD5*, *METTL6*, *CPT1A*, *DTX3*, *MRPS23*, *EIF2S2*, *EIF6* and *SLC2A10*) were found to be essential for cell proliferation, and were amplified in these patients [82]. As a Notch-dependent regulator of cell invasion and migration, eIF6 could inhibit lymphomagenesis and tumor progression [80] (Figure 8). In addition to regulating translation and Notch signaling [83], eIF6 has been reported to link integrin-β4 to the intermediate cytoskeleton [84], resulting in downstream activation SRC signaling [82] (Figure 2).

## 3. Eukaryotic Translation Initiation Factors Dependencies in Breast Cancer

**Figure 9:**
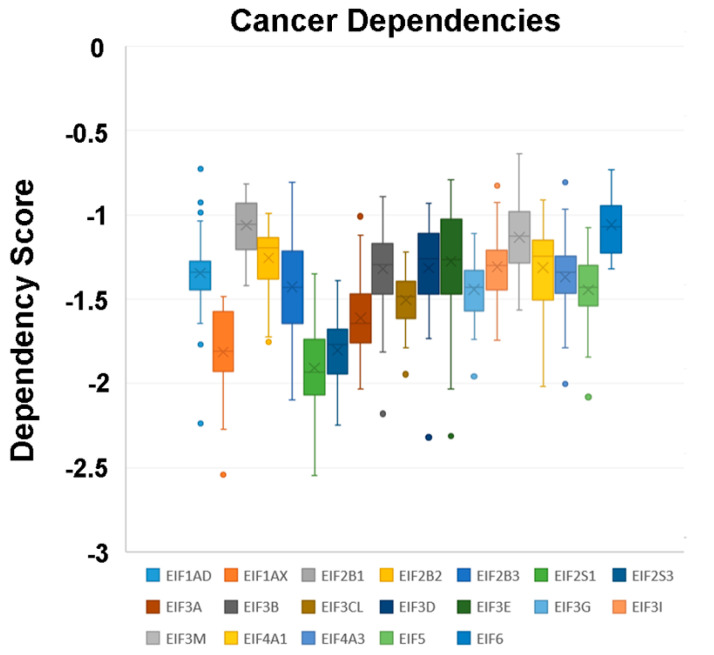
Cancer dependencies for the eIFs as taken from the depmapportal (https://depmap.org/portal/). Only eIFs exhibiting mean dependencies lower than −1 are shown.

**Figure 10:**
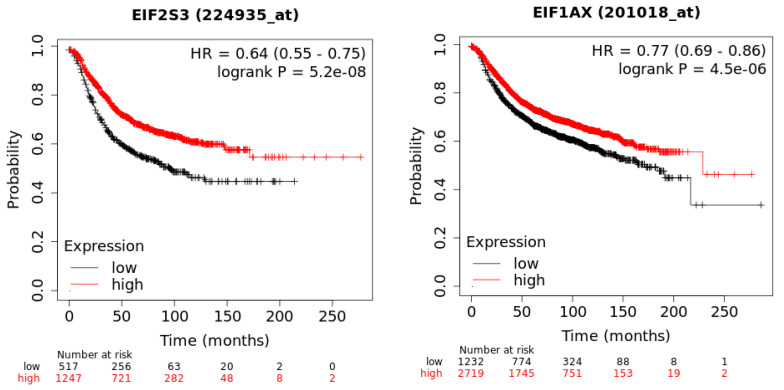
KM-plotter result for eIF2S3 and eIF2S3 for relapse-free survival in breast cancer.

In a recent publication, a strategy to identify putative drug targets based on the genetic manipulation of cancer cell lines has been developed [85,86]. For this approach, cell lines have either been manipulated by systematic RNA-interference or CRIPR/CAS9 knockdown of genes. In order to assess the importance of eIFs for breast cancer cell lines, we have downloaded the data for the CRISPR/CAS9 knockdown for the eIFs available. A cancer dependency score of zero indicates that a gene is not essential for a cell–line and a score of −1 represents the median of all common essential genes. We therefore selected eIFs that have a mean dependency score in breast cancer cell lines smaller than −1 (Figure 9). The lowest score was found for eIF1AX, eif2S1 (eIF2α), eIF2S3 (eIF2γ) and eIF3a. The importance of eIF2α and eIF3A for breast cancer is clear from the literature. Interestingly for eIF1AX and eIF2γ, published data are scarce and deduced from the cancer dependency data—these genes should be investigated in more detail. Survival data from the KMplotter indicate, indeed, a strong prognostic effect for eIF2S3 and eIF1AX [87] (Figure 10).

## 4. Discussion

eIFs regulate the assembly of the functional ribosomal complex. There are six eIF-complexes participating in translation initiation. Disorders of eIF expression by over-expression, down-regulation or phosphorylation lead to carcinogenesis or tumor progression. Disturbance of the translation initiation leads to an abnormal cellular proteome, followed by uncontrolled cell growth. This highly controlled translation initiation process has attracted the attention of researchers, leading to an increased interest in targeting cancer by modifying translation initiation. Recent research on eIFs in BC has significantly expanded our knowledge on the broad spectrum of different stimuli of mammary epithelial cells (Table 2), having an impact on protein biosynthesis.

Both eIF1 and eIF1a are required in translation initiation, by changing the ribosomal structure to facilitate mRNA-scanning, in order to allow start codon recognition. eIF1 can be affected by different types of genotoxic stress signaling.

The structure of eIF2 is more complex with regard to the heterotrimeric composition of the three subunits eIF2a, b and c. Increased phospho-eIF2α not only inhibits mRNA translation initiation, but also facilitates the translation of selected mRNAs. In chemotherapy, Trastuzumab in HER2+ BC can initiate a PKR/eIF2α-P pathway; eIF2α-P may be a potential biomarker for Trastuzumab efficacy. It is known that chemotherapy is associated with lots of side effects, limiting its use. The inhibition of eIF2α possibly suppresses BC growth and metastasis, which puts forward a new way of treating BC.

eIF3 participates in a few physiological and pathological processes. Some of the subunits emerge as new potential drug targets. eIF3a is also a potential oncogene involved in cancer occurrence, metastasis and therapy response. According to the physiological and pathological function, eIF3a represents a potential drug target. We need more efforts to improve the usability of currently available molecules or to design novel small molecule eIF3a regulators.

Among all the subunits of eIF4, eIF4E is of very high concern. eIF4E activity is regulated through the over-expression or phosphorylation of its binding protein 4E-BP1 in female BC. Phospho-4E-BP1 is a potential candidate for therapies directed towards its upstream kinase (mTOR). Moreover, the knockdown of eIF4E has also been shown to reduce BC cell migration and invasion.

As auxiliary proteins, participating in stimulating specific processes, the roles of eIF5, eIF5A and eIF5B in BC have not yet been well described during the protein translation cycle. Acting as a ribosomal anti-association factor, eIF6 has never been researched thoroughly either.

eIFs principally act as regulators of the translation initiation stage, a highly critical step, of utmost interest for targeting cancer. More detailed studies on eIFs are needed to provide further insights into the translation process in malignancies, which could provide important clues for the treatment of BC. Further studies on small molecule disruptors of eIFs or respective subunits will contribute to gaining a better understanding of the role of translational control, and will be one of the main focuses of research in the coming years.

## Figures and Tables

**Table 1:** mTOR pathway alteration (potential therapy target) in breast cancer.

Alteration	Effect on Signaling
**Gene**	
1. Activating mutations or amplifications	
*PIK3CA*	Activation of PI3K signaling
*PKB/AKT*	Activation of AKT signaling
*PDK1*	Activation of AKT signaling
*ERBB2*	Activation of ERBB2 signaling
*IGFIR*	Activation of IGFIR signaling
*FGFRI*	Activation of FGFRI signaling
2. Loss of function mutations or under-expression	
*PTEN*	Activation of PI3K signaling
*INPP4B*	Activation of PI3K signaling
*LKB1/STK11*	Activation of AKT and inhibit of TSC1/2
*PHLPP*	Activation of PI3K signaling
**Cross reacts with other pathway**	
RAS-MAPK	
SRC	
**Protein complexes**	
mTORC1	Inhibition of 4EBP1 and activation of S6K
mTORC2	Activation of AKT signaling

**Table 2:** eIF subunits and differential expression in breast cancer cell lines.

Protein	Differential Expression	Ductal Cancer	Lobular Cancer	Cell Lines	References
eIF2α	↓ (eIF2α-P↑)	√		BMECs MCF-7 SkBr3	[25,33]
eIF 3a	↑	√			[35]
eIF 3c	↓	√		BT474 MDA-MB-231	[38]
eIF3f	↓			MCF-7	[37]
eIF3h	↑			MCF-7 MDA436 SK-Br-3	[39]
eIF4a	↑			MCF-7	[64]
eIF 4b	↑			MCF-7	[64]
eIF 4e	↑	√		MDA-MB-231 MDA-MB 468 MCF-7 HMECs	[42,43,44,45,49,52,61]
eIF 4g	↑	√			[10]
eIF 5	↑	√			[75]

Abbr.: HMECs: human mammary epithelial cells; BMECs: bovine mammary epithelial cells. ↓: decrease; ↑: increase.
